# Public responses to proposals for a tax on sugar-sweetened beverages: A thematic analysis of online reader comments posted on major UK news websites

**DOI:** 10.1371/journal.pone.0186750

**Published:** 2017-11-22

**Authors:** Molly Thomas-Meyer, Oliver Mytton, Jean Adams

**Affiliations:** 1 Public Health Department, Essex County Council, Chelmsford, United Kingdom; 2 Centre for Diet and Activity Research (CEDAR), MRC Epidemiology Unit, University of Cambridge, Cambridge, United Kingdom; The Chinese University of Hong Kong, HONG KONG

## Abstract

**Background:**

Regular consumption of sugar sweetened beverages (SSBs) is associated with weight gain, type 2 diabetes, and dental caries. The UK will introduce a levy on the manufacturers of SSBs in 2018. Details will be negotiated over the next two years. How the UK public views SSB taxes is likely to be an important determinant of the content and success of the final policy. We aimed to capture the views, ideas and concerns of commenters on major UK news websites on SSB taxes.

**Methods and findings:**

We conducted a qualitative analysis of reader comments to online news coverage of one proposal for an SSB tax in the UK. 1645 comments on four articles were included. Three underpinning themes influenced support or opposition to the tax: the balance between individual responsibility and autonomy, and population need; mistrust of the intention of the proposed tax and those promoting it; and variations in the perceived complexity of unhealthy diets and obesity associated with variations in what are considered appropriate interventions. Arguments under each theme were used to justify both support and opposition in different cases.

**Conclusions:**

As the final form of the UK SSB tax is negotiated, effort should be made to address the concerns we identified. Our results suggest these efforts could usefully focus on emphasising the social and environmental determinants of diet and obesity, reinforcing the benefits of the tax to the NHS, and pitching the tax as playing into a variety of different conceptualisations of obesity.

## Introduction

In March 2016 the UK’s Chancellor of the Exchequer (Finance Minister) announced a new levy on sugar sweetened beverages (SSBs). At the time of writing, most of the details of this had not yet been confirmed, but it had been stated that pure fruit juices and milk-based drinks would be excluded, that the levy would have two tiers at over 5g and 8g of sugar per 100ml, would be payable by large manufacturers on their UK sales, and would be introduced in April 2018, following a period of consultation.[1]

Regular consumption of sugar sweetened beverages (SSBs) has been linked in systematic reviews to weight gain and increased incidence of type 2 diabetes, dental caries and obesity.[2–5] Sugar-sweetened beverages contain added caloric sweeteners often alongside few, or no, healthful nutrients. Many healthier forms of hydration exist. This makes SSBs a uniquely focused target for fiscal regulation. Systematic reviews indicate that price increases—including taxes on SSBs—could reduce consumption, but most evidence is from modelling studies.[6, 7]

The public health community largely welcomed the Chancellor’s announcement.[8] However, there were many details to be negotiated before implementation. Experiences of SSB taxes elsewhere, and of regulation of other unhealthy commodities, suggest there may be strong efforts by those with commercial interests in SSBs to influence the negotiation process.[9, 10] To make the most of the opportunity offered by the Chancellor’s announcement, the public health community needs to contribute to the consultation and ongoing discussions to ensure the final policy is evidence-based and maximises the potential benefit to the public’s health.

How the UK public views SSB taxes is likely to influence decisions about the final form of the levy, and may also play a role in successful implementation and eventual effectiveness of the levy.[11–13] Surveys and opinion polling indicate that public support for SSB taxes varies over time and place.[12, 14–28] Public health advocates seeking to develop the strongest possible SSB levy in the UK may find it useful to understand public opinion in order to build public support.

A range of surveys of public opinion and content analyses of media coverage on SSB taxes have been published, particularly from the USA.[19–26] These report support for SSB taxes ranging from 36–60% of participants, although lower proportions generally believe that such taxes will be effective in reducing consumption or obesity. A range of pro- and anti-tax arguments have been featured in previous newspaper reports. These include the benefit of such taxes to younger people who tend to be higher consumers, and society as a whole in terms of reduced healthcare costs; as well as the risk of damage to the economy from reduced sales, and the potentially regressive nature of SSB taxes.

However, few in-depth qualitative analyses are available. Qualitative studies from Australia and the UK find that pricing strategies with health-promotion intentions are generally considered by the public to be ineffective unless they achieve a very high increase in price–often quantified by participants as at least 50%.[12, 27, 29] These strategies are also only considered acceptable if revenues are spent directly on health promotion. However, there was substantial mistrust that revenues would be spent on health promotion, rather than being diverted to generic ‘government’ business. Some of these previous studies focused on health-related food pricing strategies in general, rather than SSB taxes specifically and there is little existing qualitative research focusing specifically on the acceptability of SSB taxes. Given the uniquely health damaging nature of SSBs compared to other foods, views related to SSB taxes may be different to other health-related pricing strategies.[27]

Qualitative research commonly uses transcripts of interviews or focus groups as data for analysis. One alternative source of qualitative data that is increasingly used for research purposes is on-line, user-generated content. This data requires few resources to access, and is often generated for reasons other than research–potentially reducing some biases.[30] We have previously found that findings identified from qualitative analysis of on-line, user-generated content closely matched those from more standard, resource intensive, focus group methods.[13]

We aimed to capture the views, ideas and concerns that members of the UK public commenting on popular news websites hold in relation to SSB taxes.

## Methods

We conducted a qualitative analysis of reader comments to online news coverage of one proposal for an SSB tax in the UK. The qualitative approach allows an in-depth analysis of people’s views. Unlike standard quantitative research, the intention of qualitative research is not to capture a representative sample that can produce generalizable findings, but to explore the views present about one particular phenomenon in-depth. Analysis of reader comments has previously been used to assess opinions on the acceptability of other potentially controversial public health interventions,[31] with findings reflecting those from other forms of qualitative data (e.g. focus group discussions). This suggests that using reader comments can produce ‘transferable’ findings similar to those from other data sources.[13]

We first identified one relevant ‘event’ concerning a proposed SSB tax in the UK that was widely covered by online news websites. We then identified articles covering this event published on popular UK news websites. Finally, we extracted and analysed online reader comments to these articles.

We report our methods and results in accordance with the Consolidated Criteria for Reporting Qualitative Research (COREQ; see **S1 File**).[32] Deviations from the COREQ framework reflect its focus on interview-based, rather than written, data.

### News ‘event’ of interest

We included reader responses to online articles covering publication of a report published on 29 January 2013 by a UK food and farming charity (Sustain). This called for the introduction of a 20% duty on SSBs in the UK, with revenue raised used for healthy, sustainable, food initiatives for children.[33] Although the report and press release used the term ‘duty’, it was almost universally described as a ‘tax’ in news reports and reader comments. We, therefore, use the term ‘tax’ throughout.

### Online news coverage of the ‘event’ of interest

We defined popular UK news websites as the 10 websites with the most ‘total unique visitors’ in January 2013, as reported by industry analysts comScore MMX.[34] We excluded: the website of a large producer of local news content as we expected that visitors to particular local-subsites within this would be low; sites that did not produce original content in order to avoid any duplication of news coverage; and those that were not free to access as we felt these were likely to reflect a particularly unrepresentative readership. We searched the remaining websites for news articles covering the ‘event’.

### Online reader comments

All included news articles were on websites that had facilities for reader comments. In all cases, commenters had to register and create an account before leaving a comment and their comments were identified by semi-anonymous ‘usernames’. Comments were largely unrestricted and usually only removed if they featured explicit, offensive or defamatory material. Comment sections were found at the end of articles and commenters were able to post numerous times and reference previous comments. This allowed ‘conversations’ to develop.

### Data collection and analysis

All reader comments on included news articles were downloaded on 28 April 2015. We used a modified version of the Framework Method[35] to generate themes. A review of previous, primarily quantitative, literature helped us identify preliminary themes.[12, 14–29] The lead researcher (MTM) then performed an immersive read through of all comments and identified overarching thematic areas and sub-themes. These were compared with the preliminary themes and iteratively refined and developed during re-reading. A randomised 10% of the comments included in the sample (n = 165 of 1645) were independently read by two other researchers (OM and JA). Emerging themes and sub-themes were discussed and developed. Agreed themes and sub-themes were then developed into a framework of detailed codes that were used by the lead researcher (MTM) to conduct a full and detailed coding of all comments. Illustrative, representative, verbatim (with no correction of grammar or spelling), quotations for each sub-theme were then identified and are presented in the results section. Although data saturation was reached before all comments had been coded, all comments were coded for completeness. Data analysis and coding was conducted using *NVivo for Mac* (version 10.2.1, QSR International). The background and experience of the research team is described in **S2 File**.

### Ethics and consent

Online research is relatively new, and relevant ethical principles are evolving. Commenters did not provide explicit consent to take part in research. However, comments were freely given and posted on publically available, open access websites. Conceptually, the data is similar to that from observations of behaviour in “public situations where those observed would expect to be observed by strangers”, for which the British Psychological Society advises it is not necessary to obtain informed consent.[36] Consequently, and in line with previous research,[30, 31] we believe it is ethical to use these data for research without explicit consent. As there were no specific ‘participants’ in the research, ethical approval was not required. Nonetheless, usernames and identifying details have been omitted from illustrative quotations.[37, 38]

### Data availability

The data we used can be freely accessed from the included websites.

### Study setting

A total of five websites met the inclusion criteria (see **Table 1**). Of these, four covered the event of interest—in one article each. A total of 1,645 reader comments were made on these four articles by 1158 separate usernames and were included in the analysis (see **Table 2**). The number of comments per article ranged from 17–1003. Comments ranged in length from one word to several hundred, with the majority of longer comments being from the Guardian website. All four included articles were broadly based on the press release issued by Sustain and included broadly similar content (see **Table 3**). Three articles (BBC, Guardian, Telegraph Media Group) included reactions to the proposal from other sources (e.g. the Department of Health, politicians, and the British Soft Drinks Association).

**Table 1:** The 10 most popular news websites in the UK in January 2013 and whether they met the inclusion criteria.

News Site	Total unique visitors (1000s), Jan 2013^1^	Website description	Met inclusion criteria (national news site, produces original content, free to access)
BBC	21 199	UK broadcaster with free-to-access online news platform	Yes
Mail Online	12 819	UK newspaper with free-to-access online news platform	Yes
The Guardian	11 861	UK newspaper with free-to-access online news platform	Yes
About	10 636	News aggregator	No–does not produce original content
Telegraph Media Group	9618	UK newspaper group with free-to-access online news platform	Yes
Yahoo-ABC News Network	8239	News aggregator	No–does not produce original content
Gannett Sites	6970	Local new media group	No–local news site
HPMG News	6318	News aggregator	No–does not produce original content
Independent.co.uk	5434	UK newspaper with free-to-access online news platform	Yes
The Sun Online	5226	UK newspaper with online news platform accessible via payable subscription	No–not free to access

^1^Source: comScore MMX, UK, January 2013[34]

**Table 2:** Characteristics of articles included in the analysis.

	BBC	Mail Online	Guardian	Telegraph Media Group
**Article title**	Call for soft drink sugar tax in Budget	Experts call for a 7p 'fat tax' on sugary soft drinks in bid to curb child obesity	Tax sugary drinks to boost child health, says report	Tax sugary drinks to improve health: leading organisations
**Comments, n**	1003	401	224	17
**Unique user IDs, n**	638	381	127	12
**URL**	http://www.bbc.co.uk/news/health-21228122	http://www.dailymail.co.uk/news/article-2269865/Experts-7p-fat-tax-sugary-soft-drinks-bid-curb-child-obesity.html	http://www.theguardian.com/world/2013/jan/29/tax-sugary-drinks-child-health	http://www.telegraph.co.uk/news/health/news/9832016/Tax-sugary-drinks-to-improve-health-leading-organisations.html

**Table 3:** content of included articles.

	BBC News	Mail Online	Guardian	Telegraph Media Group
**Pro tax arguments featured**				
Negative health consequences of SSB consumption	yes	yes	yes	yes
Positive health consequences by reducing SSB consumption	yes	yes	yes	yes
Revenue generated to be used to help children	yes	yes	yes	yes
Analogy with tobacco duties	yes	yes	yes	yes
Government's role to implement public health policies	yes	yes	yes	yes
SSBs are an appropriate target	yes	yes	yes	yes
Impact of diet related health problems on society/NHS	yes	yes	yes	yes
Other countries have SSB taxes	no	yes	no	no
**Anti tax arguments featured**				
Voluntary industry code is in place	yes	no	no	yes
SSBs are not an appropriate target	yes	yes	yes	yes
SSB tax will not reduce obesity	yes	yes	yes	yes
Consumption is a matter of personal choice	no	no	no	yes
VAT is already charged on SSBs	yes	no	no	yes
Additional taxes are inappropriate during a recession	yes	no	no	yes

Characteristics of visitors to the websites where included articles were published are summarised in **Table 4**. Mail Online had a relatively even gender balance, whilst men were over-represented amongst visitors to other sites. Visitors to Telegraph Media Group had a slightly older profile, and were more likely to be in higher social grades than visitors to other sites. Characteristics of those who commented on included articles are not known.

**Table 4:** Characteristics of visitors to websites of included articles in 2013.

	BBC News	Mail Online	Guardian	Telegraph Media Group
**Female, %**	40	53	42	42
**Age group**				
**16-24y, %**	17	20	21	21
**25-34y, %**	25	22	36	26
**35-54y, %**	44	44	39	31
**55y+, %**	14	14	14	22
**Social grade**^1^				
**AB, %**	37	44	47	57
**C1, %**	35	27	31	18
**C2, %**	14	14	9	9
**DE, %**	14	15	13	16

^1^Social grades using the National Readership Survey classification.

AB: upper middle and middle class; C1: lower middle class; C2: skilled working class; DE: working class and lower; Source: News Consumption in the UK 2013, Kantar Mediapanel and Ofcom.[34]

## Results

**Fig 1:**
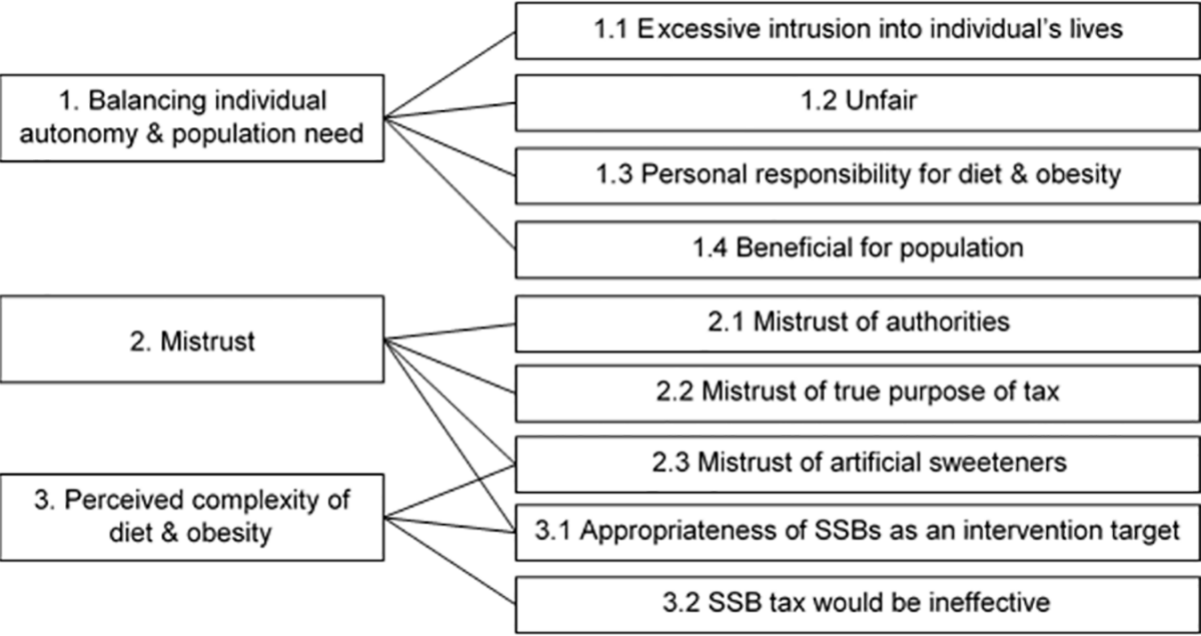
Three themes and nine sub-themes found in the data and their inter-connections.

We identified three themes, with nine sub-themes (**Fig 1**). In our detailed description of results below we have allocated each sub-theme to a theme. However, the substantial inter-connection between themes and sub-themes was a key feature. Similarly, individual comments often touched on more than one theme or sub-theme.

### Theme 1: Balancing individual autonomy and population need

The primacy of the autonomy of the individual–both specifically to make their own dietary choices, and more generally not to be overly intruded on by government and other authorities–was a key overarching theme. An SSB tax was considered unfair as it impinged on what was felt to be individuals’ right to choose to drink SSBs. In stressing the rights of the individual, commenters often blamed obesity on the actions of others, particularly in terms of irresponsible personal or parenting behaviours. Those who were more supportive of an SSB tax tended to stress the population impacts of obesity particularly in terms of health care costs borne by the National Health Service (NHS) and ultimately the taxpayer, the potential health benefits, and the societal benefits from additional government spending of revenue raised.

#### Sub-theme 1.1: Excessive intrusion into individual’s lives

Many commenters perceived the proposed tax as an excessive level of interference in their lives and choices (see **Box 1**). The extreme level of control the tax implied to these commenters was reflected in comparisons with “a tax on air” and making reference to a “totalitarian state”. Others referred to “nannying”, implying that the SSB tax was a form of patronising interference.

**Box 1. Illustrative quotes, sub-theme 1.1, ‘excessive intrusion into individuals’ lives’.**

Another attempt by 'Experts' to micro manage our already complicated lives. Leave us alone and hopefully common sense and education will prevail.

[BBC News]

Water, alcohol, cigarettes, food and now soft drinks. When is the fresh air tax coming?

[Daily Mail Online]

I don't like fizzy drinks, but taxing those who do 'for their own good' is just another step to state control, where we're employed, housed and fed by the state, while our children are brought up in state nurseries—'for our own good'. The future's Orwellian.

[BBC News]

When is this Govt going to realise we have information overload regarding what's good and what's bad for us. Why don't they just back off and accept the fact that we're human, we make choices, sometimes bad ones. But I'm an adult and I'm sick to death of being treated like a 5 year old by this nanny-state we're now living in.

[BBC News]

I am beginning to get tired of all the preaching about what we should and shouldn't ingest.

[The Telegraph Online]

#### Sub-theme 1.2: Unfair

Some commenters thought that an SSB tax would unfairly target individuals who consumed SSBs “responsibly” (see also sub-theme 1.3 below). In particular, commenters expressed a belief that the proposed tax would penalise their own behaviour which they were able to take responsibility for, when blame and punishment should more appropriately be apportioned to “irresponsible” others (see **Box 2**).

**Box 2. Illustrative quotes, sub-theme 1.2, ‘unfair’.**

I drink fizzy drinks once or twice a week, im 12 stone, and I pay over £100 in taxes per week thats before i spend anything. why should I have to pay more taxes because someone can't control their eating habits? I know people who overeat and are obese as a result and suffer with heart problems, diabetes and other problems, those people should be made to pay, not me…

[BBC News]

I drink a lot of fizzy drinks but I'm a healthy size and not over weight, BECAUSE I BURN IT OFF! Stop putting taxes on things, I pay enough taxes, takes the money off of benefits instead

[Daily Mail Online]

This is simply another tax on the poor. Bring down the price of healthy foods and then see if there is a decline in obesity. Seems this government is making sure there is a HUGE divide between the rich and poor.

[Daily Mail Online]

many people don't even look at the price when they buy the essentials, and soft drinks are for many people one of the essentials. All a tax will do is take even more money away from the working class

[The Guardian]

Others described an SSB tax as unfair because they felt the effects would fall disproportionately on those living on lower incomes. Lower income groups were perceived to be both higher SSB consumers and less able to afford increased costs. This dual effect was considered to contribute to a fracturing of society along income grounds.

#### Sub-theme 1.3: Personal responsibility for diet and obesity

Food and drink choices were often cast as the responsibility of individuals, and not something that should be of concern to society or government. Commenters in this theme often used what they framed as their own “responsible” behaviour as a model for others to follow (see **Box 3**). Typically commenters would report consuming SSBs themselves in “moderation” and balancing this with physical activity in order to maintain a healthy weight. This led to a conclusion that those who were not a healthy weight must either over-consume or under-exercise. Similar arguments were made about the parents of overweight children. Some commenters felt particularly strongly that they should not have to pay for other peoples’ “irresponsible” behaviours either in the form of the proposed SSB tax or through wider taxation to fund NHS costs. This was a further reason why an SSB tax was seen as unfair (see sub-theme 1.2). Whilst most comments within this sub-theme viewed an SSB tax negatively, arguments concerning a failure of personal responsibility were also used in support of a tax: if people are unable to take responsibility for their own behaviour they must “face the consequences”.

**Box 3. Illustrative quotes, sub-theme 1.3, ‘personal responsibility for diet and obesity’.**

… I limit my consumption of it [SSBs]. A few cans a week haven't made me fat or sickly. … Our diet over the past few decades has become dreadful. Speak for yourself. My diet is OK.

[The Guardian]

If youre fat, its your own fault and its YOUR responsibility to do something about it. Not the NHS Not the Govt Not the tax payer … Take some responsibility and put down the fork!

[BBC News]

… some people arguing against this sensible proposal sound a bit like those gun fanatics in America quoting the second amendment. If people insist on being too thick to take responsibility for themselves, then they have to accept that such behaviour comes with a price tag.

[BBC News]

Yet again, the rest of society has to pay more for those who are unable to control what they put in their mouth. You have to be pretty stupid if you haven't heard the news—fizzy drinks, processed food, cakes, chocloate, takeaways, ready meals etc when consumed day in day out will make you fat!

[Daily Mail Online]

What makes them fat is not the lack of taxation on fizzy pop, it is the fact that they are lazy gluttons

[The Guardian]

#### Sub-theme 1.4: Beneficial for the population and the NHS

Those who were supportive of the proposed SSB tax often invoked arguments related to the wider benefit to society, particularly in the longer term (see **Box 4**). A number of suggestions were made for how tax revenues could be spent to benefit society as a whole–particularly via additional financial support for the NHS. It was also proposed that a tax would encourage drinks manufacturers to reformulate SSBs to reduce the sugar content. Some commentators suggested a higher tax than proposed, or extending it to cover a wider range of foods and drinks. Commenters also identified a need for additional interventions beyond an SSB tax. Whilst some commenters felt that the beneficial effects of an SSB tax were likely to be small, they also felt the NHS was so important, and so much in need of support, that this would justify even small health gains from an SSB tax.

**Box 4. Illustrative quotes, sub-theme 1.4, ‘beneficial for the population’.**

Seems like a great idea, if it'd work. It would encourage the drink producers to reduce the sugar, the sugar drinkers to consume less, and the money it makes go back into either supporting a health service overburdened by caring for the sugar drinkers or increasing the likelihood of fewer sugar drinking kids in the future. Win-win-win!

[BBC News]

It doesn't solve anything but some of the tax will go towards the NHS and help pay for the treatment of obesity.

[Daily Mail Online]

Use the cash to pay for 'free' dentistry (NHS) for all.

[The Telegraph Online]

If a tax helps that then so be it, it's worth the extra cost (and will save us loads in the future). More needs to be done still, but we simply cannot let this ticking time bomb to continue as is.

[The Guardian]

I think that 20p a litre extra for a soft drink is not a large amount of tax to pay. The money raised would be sufficient to make a difference to funding free fruit for school meals. It is a good idea.

[BBC News]

### Theme 2: Mistrust

Underlying many of the comments rejecting an SSB tax was a strong sense of mistrust of the government, politicians, the SSB industry, and public health experts. One of the main sources of this mistrust was a belief that the proposed tax was primarily a means for raising revenue that would be inappropriately used by government. There was a widespread rejection of the idea that an SSB tax was truly intended to improve population health. There was also mistrust of artificial sweeteners, and concern that the potential unintended consequence of a tax leading to increased consumption of artificial sweeteners had not been well considered.

#### Sub-theme 2.1: Mistrust of individuals and organisations in positions of authority

Many commenters interpreted the proposed SSB tax as government-led (it was a proposal from the charity Sustain). This led to suggestions that the proposal demonstrated that the government was “out of touch” with the population (see **Box 5**).

**Box 5. Illustrative quotes referring to sub-theme 2.1, ‘mistrust of authorities’.**

Yes, of course, everyone should eat healthily, but then healthy food is more expensive. This wretched government is so out of touch I doubt many of them can think intelligently enough to realise that if you cut welfare so aggressively, people will opt for the food they can afford.

[The Guardian]

THEY HAVE DONE IT—TAX SCHOOL CHILDREN, How much lower can they go, I suppose this tax will make enough to give the MP's the over the top pay rises they want.Crooks the lot of them

[Daily Mail Online]

Bigwigs from the food industry get away with putting all kinds of crap into our food thats the cause of most of todays problems?

[The Telegraph Online]

Picking easy targets that are percieved to be bad for you is just too easy, These Health bodies should start doing some hard work and look at the whole picture, not a blinkered one

[BBC News]

How many of these "experts" struggle with their grocery bill? I`m sick and tired of hearing "experts" calling for a rise in the cost of living—it`s far too high in this country as it is. It would make more sense to me if there were a corresponding drop in the price of more healthy food.

[Daily Mail Online]

A similar mistrust was expressed for the SSB and wider food industry. The food industry was perceived as being at fault for producing SSBs and as holding undue political power and influence.

For those who identified Sustain as the source of the proposal, mistrust extended to Sustain, and other experts–including public health experts. The credibility of both Sustain, and these experts, was questioned because the advice they offered was considered patronising and simplistic compared to the perceived complexity of diet and obesity.

#### Sub-theme 2.2: mistrust of true purpose of tax

Mistrust of individuals and organisations in positions of authority often overlapped with cynicism about the intention of the proposed SSB tax (see **Box 6**). Commenters thought the true aim of the tax was to raise revenue, rather than reduce SSB consumption. The perceived ineffectiveness of the tax was used as justification to support this idea (see theme 3.2). Sometimes these claims were associated with suggestions that the government would use revenue raised from the tax inappropriately. To reinforce this, commenters often drew comparison with the 2009 scandal of UK Members of Parliament over-claiming expenses.[39]

**Box 6. Illustrative quotes, sub-theme 2.2, ‘mistrust of true purpose of tax'.**

Anyone who believes for a minute that any revenue raised from this tax would be specifically allocated to free school meals and dietary education is, I'm afraid, being rather naive. Like everything else it will just disappear into the Treasury's piggy bank no doubt to be used at a later date to fund tax reductions for the "wealth creating" high-sugar food and drinks industry.

[The Guardian]

Any tax less than a pound per bottle/can is meaningless. A 7p hike won't stop ANYONE buying ANYTHING, it will just go into the government's coffers and thus they will be making money out of the problem just like they do with cigarettes. Disgraceful.

[Daily Mail Online]

We want more of your money but will disguise it as a "health concern"—are people really so gullible. You could educate parents and stop them buying sugary soft drinks and high sugary sweets for the kids. …but that does not suck money out of people, tax'em.

[The Guardian]

Sounds OK as long as every single penny raised is ring-fenced and spent on providing fruit in schools and other worthwhile causes. What would actually happen is that the money raised will drop into the ocean of tax revenues and be irresponsibly squandered.

[BBC News]

#### Sub-theme 2.3: Mistrust of artificial sweeteners

Commenters frequently assumed that consumption of artificially sweetened drinks would increase as a result of the tax. This consequence was felt to be due both to consumers switching from sugar sweetened to artificially sweetened products, and manufacturers reformulating existing drinks to replace sugar with artificial sweeteners. There was a strong mistrust of artificial sweeteners and a variety of potential health-damaging effects of artificial sweeteners were described (see **Box 7**). In some cases, mistrust of artificial sweeteners fuelled mistrust of public health ‘experts’, who were felt to endorse the view that artificial sweeteners were safe (see theme 2.2).

**Box 7. Illustrative quotes referring to sub-theme 2.3, 'mistrust of artificial sweeteners'.**

I would much prefer to feed my children a sugary drink then take them out to play in the park and burn it off then so-called diet drinks filled with neurotoxins like Aspartame that does Lord-knows-what to their developing brains.

[BBC News]

As a nutrition and health professional, I would be broadly in favour, but I would NOT want people to go away with the idea that having "diet" fizzy drinks instead would be good for you.

[The Guardian]

There has been quite a bit of research that shows that even "diet" drinks are fattening as the sweeteners fool the body into expecting something sweet. Insulin levels rise accordingly and then when the body detects no sugar the craving starts.

[Daily Mail Online]

Don't worry about the sugar content, it's the aspartame and other artificial sweeteners one needs to worry about.

[The Telegraph Online]

### Theme 3: Perceived complexity of diet and obesity

Whether the causes of diet and obesity were perceived to be complex or simple appeared to be an important determinant of perceived effectiveness of an SSB tax. Perceived effectiveness of the tax was, in turn, an important determinant of acceptability. For some commenters obesity was simple: the result of a lack of self-control leading to overconsumption and insufficient physical activity. For these commenters, an SSB tax was considered unlikely to be effective because it does not address issues of self-control. For others, obesity was perceived as a complex problem with many and varied determinants. In these cases, an SSB tax was considered too simplistic and likely to fail on its own because it addresses only one small part of a complex problem.

#### Sub-theme 3.1: Appropriateness of SSBs as an intervention target

Perceived acceptability of an SSB tax often hinged on whether SSBs were felt to be a suitable target for intervention (see **Box 8**). Many commenters did not believe that SSBs were a sufficiently important cause of obesity to merit the perceived intrusiveness of a tax. For a few, there was a total rejection of SSBs as a cause of obesity–often because they felt they had no personal evidence of a link between SSB consumption and weight gain.

**Box 8. Illustrative quotes referring to sub-theme 3.1, 'appropriateness of SSBs as an intervention target’.**

Well, if your only justification is to be consumed year round, I guess you'll be taxing mars bars, kitkats, lollies, ice cream, all chocolate bars, cups of tea with 2+ lumps of sugar, right? They're all loaded with yummy sugar. Where does your arbitrary taxing madness end?

[BBC News]

I've drank soda all my life and I've never been overweight.

[Daily Mail Online]

Where ARE all these fat kids?? Just been to my grandson's school assembly. 400 children present and only five could be classed as fat, using one eyes rather than these ridiculous charts which have been shown to be incorrect for children's BMIs.

[Daily Mail Online]

Soft drinks are the single most easily removable, ultra high caloric density factor in the obese diet.

[The Guardian]

It's as dangerous as cigarettes and to the youngest of children. Tax it and tax it again.

[BBC News]

Conversely, supporters of the tax identified SSBs as an appropriate target for intervention because they perceived them as both harmful to health and non-essential. Some also felt that SSBs were an appropriate target because they were perceived to be disproportionately consumed by children.

#### Sub-theme 3.2: SSB tax would be ineffective

Acceptability was often dependent on whether an SSB tax was perceived to be effective in reducing obesity (see **Box 9**). Many commenters thought it would not be. Some suggested that human desire for sugar was very strong and that this would not be affected by price. Others thought consumers would simply adapt their behaviour to avoid the price increase–for example by bulk buying preferred brands or seeking out cheaper alternative brands. Others felt the price increase associated with the proposed tax would be too small to be effective. This led to some, who were supportive of the tax, to propose a much higher level. Finally, some felt that external factors (such as manufacturers manipulating wholesale prices) would undermine the tax’s effectiveness.

**Box 9. Illustrative quotes referring to sub-theme 3.2, ‘SSB tax would be ineffective’.**

Smart people who are well educated about nutrition still make the wrong choices when emotional eating is involved. Plus the human brain is stupid, as soon as you tell us what is bad and what we can't have, we want it more and or feel guilty for "indulging" in it.

[Daily Mail Online]

Those that indulge in things to excess will continue to do so regardless by cutting down on expenditure elsewhere.

[BBC News]

7p is not enough, it should be much higher to make a difference/make people think twice about buying sugar laden drinks!!!

[Daily Mail Online]

If government taxes soda, all that means is that brands will drop soda prices so as not to deter consumers. I imagine therefore that the prices of "healthy alternatives" (now owned by the soda brands BTW) will go up to compensate.

[The Guardian]

## Discussion

We identified three underpinning themes that influenced support or opposition to a proposed SSB tax in the UK: the balance between individual responsibility and autonomy and population need; a mistrust of the intention of the proposed tax and those promoting it; and variations in the perceived complexity of unhealthy diets and obesity leading to variations in what were considered appropriate responses. There was strong support for the NHS, and a feeling that the NHS was under financial threat. For some, this justified strong action, such as an SSB tax. There was no simple relationship between themes and support or opposition to the tax, with arguments under most themes being used to justify both support and opposition in different cases.

The general opposition found to an SSB tax reflects concurrent UK opinion surveys.[12, 14, 15] Whilst more recent UK opinion polls find around 50% support for an SSB tax,[17, 18] we do not think the views expressed in relation to SSB taxes are likely to have changed recently.

Our findings that diet and obesity were often felt to be a matter of personal responsibility, reflect previous findings.[12, 15, 23, 40] While previous work describes majority support for the idea that being overweight is due to a lack of individual willpower, a large proportion of people also believe that the prevalence of unhealthy foods in the environment has an important influence–and these beliefs are not necessarily mutually exclusive.[15, 40] This suggests that the ‘social determinants of health’[41] model is recognised by the public and may drive some of the supportive comments found.

The population need that was set against concerns for individual autonomy often focused on the NHS–which was, as previously, perceived to be under financial threat.[42, 43] Differences in the perceived acceptability of other public health interventions in the UK compared to other high-income countries with insurance-based healthcare systems have also been attributed to the differential context of the NHS.[44, 45]

Our theme of mistrust was wider than previously described,[12, 20, 21, 29] extending beyond central government to other sources of authority. In particular, there was a concern about the unintended consequence of SSB taxes on artificial sweetener consumption. This has been described previously.[12, 27] Further work may be required to establish how generalised a concern this may be across the population.

Whilst Sustain described the proposal as a “duty”, the term “tax” was much more commonly used in articles and comments. Most people are unlikely to consider a duty (i.e. an excise tax) to be qualitatively different from other taxes and it may not be helpful, or necessary for advocates, to avoid words such as ‘tax’.

Like other work, we found that an SSB tax was perceived by many to be ineffective.[23, 27, 29] This may explain much of the opposition identified. This perceived ineffectiveness was expressed both by those who felt the ‘problem’ of obesity was simple, and by those who felt it was complex. In its simple form, obesity was seen as a failure of individual responsibility and willpower. In its complex form, obesity was regarded as a result of multiple and interrelated social and environmental factors. It was felt a tax would do little to address either of these perceived causes. This ‘lay epidemiology’[46] should be taken into account when communicating the value of potential solutions.

### Strengths and limitations

As the data was collected after the last comment had been posted, there was no researcher interference in the process of commenting. This may increase the likelihood that commenters expressed their true feelings. Conversely, the anonymity of the internet may mean commenters do not feel they have to be truthful or polite.[47] It is also possible that commenters may have been paid by various parties to make comments. It is difficult to know the extent of this practice, or impact on our findings. Indeed, we know relatively little, if anything, about the people who made the comments included. They may not be representative of either the UK population as a whole or the readers of the websites or articles included.

The lack of researcher interference in the data means that we were unable to probe commenters’ reasoning and meaning. Despite this, the complexity of the themes and sub-themes we identified suggests that comments were not necessarily superficial.

Qualitative research does not intend to capture representative samples or produce generalisable findings. Instead, the intention is to generate an in-depth understanding of a particular phenomenon and explore ‘transferability’ to other contexts.[48] To this end, we focussed on popular and freely accessible news websites. The similarity of our findings to those of previous researchers (reviewed above) who have used different methods to study similar topics increases our confidence in the veracity and transferability of our findings.

Whilst our results are based on comments to only four articles in response to only one ‘event’, more than 1000 comments were included and we achieved thematic saturation, indicating that our sample size is likely to be appropriate for the research aim. Commenters were unlikely to be statistically representative of the UK population as a whole, although this is not a standard concern of qualitative research. Whilst it may only be those with the strongest views who comment online,[49] it is perhaps also the loudest voices that have most effect on policy makers. This increases the value and relevance of our findings to public health policymakers.

Our data are cross-sectional and we can not necessarily be clear either what determined the views expressed, nor what the key drivers to changing these views might be.

### Implications for policy, practice and research

Additional quantitative and qualitative work in the UK should confirm our results and explore determinants of opinions, and drivers of opinion change. Additional work to understand the ideas and concerns of other stakeholders on SSB taxes would provide a more complete picture. Since media coverage interacts with, reflects and drives public opinion, an analysis of UK news coverage would also be instructive.[50]

Particular to the UK context, we found that supporters of an SSB tax often discussed this in terms of the potential benefits to the NHS. In the UK, support for the NHS could be further used to build support for public health interventions, including an SSB tax.

The Chancellor’s announcement stated that revenues from the new UK SSB levy would be ring-fenced for school breakfast clubs and school sports[1] and previous research suggests this will be important for maximising public acceptability.[12, 27, 29] However, our data suggest there is likely to be public scepticism that direct diversion of funds will occur. Further efforts may be required to ensure that the use of revenues raised by an SSB tax is transparent.

Our finding that responses to an SSB tax were related to perceptions about causes of obesity, coupled with previous endorsements of environmental determinants of obesity,[15, 40] suggest that emphasising the social and environmental determinants of diet and obesity may help build support for SSB taxes. Proponents may also find it helpful to clearly position such a tax within both the simple and complex conceptualisations of obesity. In particular, framing an SSB tax as both supporting willpower and as one small part of a complex solution.

The concern amongst commenters that an SSB tax would increase consumption of artificial sweeteners may also need to be addressed if it is found to be a generalised concern across the population. Rather than entering into debates about the safety of artificial sweeteners, it may be better to emphasise that water and unsweetened teas and coffee are healthy alternatives to SSBs. Finally, the public health community may find it instructive to consider potential responses from the SSB industry to the Chancellor’s announcement. If the SSB industry wishes to avoid a strong SSB levy, they may seek to build public opinion in this direction by emphasising the perceptions that a tax is intrusive, unfair and regressive; that individuals bear personal responsibility for diet and obesity; and by questioning the potential effectiveness of an SSB tax.

## Conclusion

As the UK moves from the announcement of an SSB levy through consultation and refinement, the public health community should seek to address outstanding public concerns in order to ensure successful and strong implementation. Our work suggests this could be, at least partly, achieved through: emphasising the social and environmental determinants of diet and obesity, reinforcing the potential benefits to the NHS of both health improvement and revenue raised, and clearly explaining how an SSB tax fits with lay conceptualisations of the causes of unhealthy diets and obesity.

## Supporting information

S1 File Completed COREQ checklist. (DOCX)

S2 File Research team and reflexivity. (DOCX)
