# Supplementary material for: Public responses to proposals for a tax on sugar-sweetened beverages: A thematic analysis of online reader comments posted on major UK news websites
Source: PLoS One. 2017 Nov 22;12(11):e0186750. doi: 10.1371/journal.pone.0186750 (PMC5699796; doi:10.1371/journal.pone.0186750)
Supplement: S2 File — (DOCX) [file pone.0186750.s002.docx]

**S2 File**

MTM completed the research as part of an MPhil in Public Health during postgraduate training in public health. MTM also has degrees in medicine (MBBS), English Literature (MA Hons) and Journalism (MSc). She completed training in qualitative research as part of her MPhil, but this was her first experience of qualitative research in practice. OM is nearing completion of postgraduate training in public health and a PhD. He holds degrees in medicine (BM BCh) and public health (MPH). He has previously published work on the potential effects and evaluation of food taxes.[1-7] This was his first experience of qualitative research. JA is a public health researcher with experience across a range of domains and methods. She holds degrees in health psychology (BMedSci and MSc), medicine (MBBS), and epidemiology and public health (PhD). She has not previously published in the area of SSB taxes, but has conducted previous qualitative research.[8-14]

**References**

1. Mytton OT, Clarke D, Rayner M. Taxing unhealthy food and drinks to improve health. BMJ. 2012;344. doi: 10.1136/bmj.e2931.

2. Mytton OT, Eyles H, Ogilvie D. Evaluating the Health Impacts of Food and Beverage Taxes. Current Obesity Reports. 2014;3(4):432-9. doi: 10.1007/s13679-014-0123-x.

3. Mytton O, Gray A, Rayner M, Rutter H. Could targeted food taxes improve health? J Epidemiol Community Health. 2007;61(8):689-94.

4. Nnoaham KE, Sacks G, Rayner M, Mytton O, Gray A. Modelling income group differences in the health and economic impacts of targeted food taxes and subsidies. Int J Epidemiol. 2009:dyp214.

5. Briggs AD, Mytton OT, Kehlbacher A, Tiffin R, Rayner M, Scarborough P. Overall and income specific effect on prevalence of overweight and obesity of 20% sugar sweetened drink tax in UK: econometric and comparative risk assessment modelling study. 2013.

6. Briggs AD, Mytton OT, Madden D, O’Shea D, Rayner M, Scarborough P. The potential impact on obesity of a 10% tax on sugar-sweetened beverages in Ireland, an effect assessment modelling study. BMC Public Health. 2013;13(1):1.

7. Mytton O. Time for a sugary drinks tax in the UK? Journal of Public Health. 2014:fdu033.

8. Adams J, Halligan J, Burges Watson D, Ryan V, Penn L, Adamson AJ, et al. The Change4Life Convenience Store Programme to Increase Retail Access to Fresh Fruit and Vegetables: A Mixed Methods Process Evaluation. PLoS One. 2012;7(6):e39431. doi: 10.1371/journal.pone.0039431.

9. Giles E, Holmes M, McColl E, Sniehotta F, Adams J. Acceptability of financial incentives for breastfeeding: thematic analysis of readers' comments to UK online news reports. BMC Pregnancy Childbirth. 2015;15(1):116. Epub 2015/05/20. doi: 10.1186/s12884-015-0549-5. PubMed PMID: 25982305.

10. Giles E, McColl E, Sniehotta F, Adams J. Acceptability of financial incentives and penalties for encouraging uptake of healthy behaviours: focus groups. BMC Public Health. 2015;15(58):doi:10.1186/s12889-015-1409-y.

11. Goffe L, Penn L, Abraham C, Adams J, Adamson A, Araujo-Soares V, et al. Interventions to promote healthier takeaway, pub and restaurant (out-of-home) food: A qualitative study of intervention deliverers’ experience In preparation. 2015.

12. Halligan J, O’Brien N, Purves R, Becker F, Goffe L, Brown H, et al. Research to support the evaluation and implementation of adult cooking skills interventions in the UK: pilot RCT with process and economic evaluations. Final project report. London: Public Health Research Consortium, 2015.

13. McNaughton RJ, Adams J, Shucksmith J. Acceptability of financial incentives or quasi-mandatory schemes to increase uptake of immunisations in preschool children in the United Kingdom: Qualitative study with parents and service delivery staff. Vaccine. 2016. doi: <http://dx.doi.org/10.1016/j.vaccine.2016.03.009>.

14. Mills S, Brown H, Wrieden W, White M, Adams J. Factors influencing cooking behaviour and decisions not to cook in UK adults: qualitative interview study with photoelicitation. in preparation. 2016.
